# Phylotypic Characterization of Mycobionts and Photobionts of Rock Tripe Lichen in East Antarctica

**DOI:** 10.3390/microorganisms7070203

**Published:** 2019-07-18

**Authors:** Merry Sailonga Faluaburu, Ryosuke Nakai, Satoshi Imura, Takeshi Naganuma

**Affiliations:** 1 Graduate School of Biosphere Science, Hiroshima University, 1-4-4, Kagamiyama, Higashi-hiroshima, Hiroshima 739-8528, Japan; 2 Applied Molecular Microbiology Research Group, Bioproduction Research Institute, National Institute of Advanced Industrial Science and Technology, 2-17-2-1, Tsukisamu-higashi, Toyohira Ward, Sapporo 062-8517, Japan; 3 National Institute of Polar Research, Research Organization of Information and Systems, 10-3, Midori-cho, Tachikawa, Tokyo 190-8518, Japan; 4 SOKENDAI (The Graduate University for Advanced Studies), 10–3, Midori–cho, Tachikawa, Tokyo 190–8518, Japan; 5 Graduate School of Integrated Sciences for Life, Hiroshima University, 1-4-4, Kagamiyama, Higashi-hiroshima, Hiroshima 739-8528, Japan

**Keywords:** lichens, symbiosis, mycobionts, photobionts, chloroplasts, cyanobacteria

## Abstract

Saxicolous rock ripe lichens that grow on rocks in the East Antarctic fellfields were sampled for phylotypic characterization of its constituent mycobionts (fungi) and photobionts (algae and cyanobacteria). The rock tripe lichen-forming fungal and algal phylotypes were classified under the common lichen-forming genera of ascomycetes, namely, *Umbilicaria*, and green algae, namely, *Trebouxia* and *Coccomyxa*. However, phylotypes of the green algal chloroplasts and the lichen-associated cyanobacteria showed unexpectedly high diversity. The detected chloroplast phylotypes were not fully affiliated with the green algal genera *Trebouxia* or *Coccomyxa*. The predominant chloroplast phylotype demonstrated maximum resemblance to *Neglectella solitaria*, which is neither a known Antarctic species nor a typical lichen photobiont. Another dominant chloroplast phylotype belonged to the atypical Antarctic green algae family. Cyanobacterial phylotypes were dominated by those affiliated with the *Microcoleus* species rather than the well-known lichen-associates, *Nostoc* species. The occurrences of these *Microcoleus*-affiliated cyanobacterial phylotypes were specifically abundant within the Yukidori Valley site, one of the Antarctic Specially Protected Areas (ASPA). The ASPA site, along with another 50 km-distant site, yielded most of the cryptic diversity in the phylotypes of chloroplasts and cyanobacteria, which may contribute to the phenotypic variability within the rock tripe lichen photobionts.

## 1. Introduction

Lichens are composite organisms that arise from an association between filamentous fungi and photosynthetic organisms, commonly termed as mycobionts and photobionts, respectively, which has often been regarded as a good example of mutualistic symbiosis. Thus far, nearly 17,000–20,000 species of fungi, which account for approximately 20% of the extant species of fungi, are capable of lichenization [1]. It is well known that approximately 90% of lichens have less diverse green algal photobionts compared to the filamentous fungal partner, of which the two major genera include *Trebouxia* and *Trentepohlia*, along with the major cyanobacterial genus *Nostoc* [2]. Approximately 85% of lichens have green algal photobionts, or chlorobionts; 10% have cyanobacterial photobionts, or cyanobionts; 3% have both photobionts, i.e., chloro- and cyano-bionts, to form tripartite (three membered) lichens; and the remaining 2% have brown or golden algae [3]. Lichens may have endolichenic or lichenicolous fungi, i.e., fungi present inside or on the surface of lichens, respectively. These fungi act as hidden potential producers of bioactive substances, such as detoxicants against anti-fungal substances from the lichen-forming fungi, antioxidants and lipase-/amylase-inhibitors, or necrotrophic/biotrophic parasites [4,5,6].

The presence of yeasts on lichens may act as possible third and fourth symbionts [7,8], suggesting that quadripartite or pentapartite (three fungi and one or two photobionts) is a common form of lichens. Therefore, lichens may be viewed as a multipartite micro-ecosystem, which harbors intricate biological and biogeochemical processes [9,10,11]. Lichens are also recognized as specific combat zones, composed of algal slaves and fungal exploiters, which are also characterized as niche seekers or take-over specialists, thus resulting in profound changes in the lichen microbiomes [12,13].

As lichens have been broadly termed as mutualistic, they are successful colonizers in dominating the vegetation of over 6% of the land surface of the Earth [14]. The wide distribution and dominance of lichens is owed to its high adaptability and acclimatization to the different environmental conditions as a result of the phenotypic plasticity in thalli structure, respiration, photosynthesis, and tolerance to dryness [15,16,17,18]. Additionally, selection and switching of photobionts contribute to an increased adaptability [5,19,20,21,22] that would eventually allow the lichens to survive and thrive in the harshest of environments such as Antarctica. In Antarctica, approximately 400 species of lichens [23] have been studied for their diversity, ecophysiology, and photobiont diversity, which may eventually respond to environmental gradients and climate changes [24,25]. It is interesting to note that in an Antarctic-wide survey, 141 phylotypes of lichens were shown to be affiliated with the green algal genus *Trebouxia* [26].

Lichens, as a micro-ecosystem in general, would be more versatile and adaptable with the involvement of bacteria in its symbiotic association. Bacteria would aid in nitrogen acquisition by N_2_-fixation, phosphate mobilization, hydrolytic activities, and production of a hormone (indole-3-acetic acid), and lichens in turn would profit from the bacteria by increasing their life expectancy [27,28,29,30]. In this study, we conducted phylotypic characterization of the mycobionts and photobionts (algae and cyanobacteria) of rock tripe lichens in Antarctica. Interestingly, during characterization, we encountered unexpected phylotypic diversity of algal chloroplasts and cyanobacteria found in these rock tripe lichens. It is discussed that there might be a possible cryptic diversity of chloroplasts and cyanobacteria against less diverse fungal and algal phylotypes in the rock tripe lichens found in Antarctica.

## 2. Materials and Methods

### 2.1. Collection of Rock Tripe Lichen Samples from Antarctica

**Figure 1:**
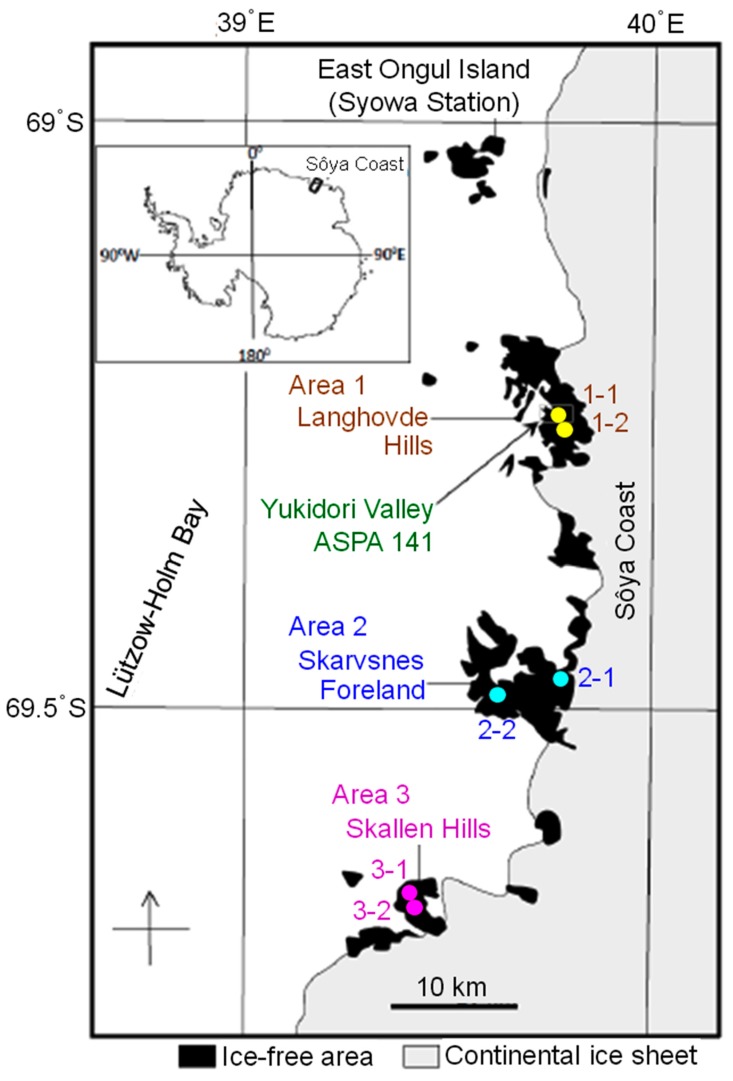
Location of sampling sites along the east coast (Sôya Coast) of Lützow-Holm Bay, near Syowa Station in Queen Maud Land, East Antarctica. Sites 1-1 and 1-2 are located in area 1, Langhovde Hills; sites 2-1 and 2-2 in area 2, Skarvsnes Foreland; and, sites 3-1 and 3-2 in area 3, Skallen Hills.

Samples of rock tripe lichens were collected between January and February 2011, during the 52nd Japanese Antarctic Research Expedition, at six different sites in the ice-free rocky fellfields, along the east coast (Sôya Coast) of Lützow-Holm Bay, near Syowa Station, in Queen Maud Land, East Antarctica (Figure 1; Table 1). The first site (area 1), Langhovde Hills (about 20 km south of the Japanese Syowa Station), was located in the northernmost part of the full studied field, and the sites were therein named as 1-1 and 1-2. The site named 1-1 was within the Antarctic Specially Protected Area (ASPA) 141, Yukidori Valley, and it is the same place from where sample 1-1 was collected. The southernmost part of the studied field was area 3, Skallen Hills, about 50 km from area 1, wherein sites 3-1 and 3-2 were marked. The remaining two sites, 2-1 and 2-2, were set in Skarvsnes Foreland, located in the center of Langhovde and Skallen Hills. It is important to note that these hills and foreland are divided by glaciers.

Lichen thalli of size greater than 3 cm and growing in a ~1 m-wide colony were collected by cutting at the umbilicus attachments with flame-sterilized scalpel blades and tweezers, and were subsequently placed together in a pre-sterilized Whirl-Pak bag (Nasco, Fort Atkinson, WI, USA). One or two colonies were sampled at each site (Table 1). It is important to keep in mind that in this study, as only one sample per site was used for mycobiont and photobiont characterization, it may not necessarily represent the site. Lichen samples were kept in the dark in the field during collection, were kept frozen during transportation, and were stored at −20°C in the laboratory freezer until use.

### 2.2. DNA Extraction from the Lichen Thalli

Approximately 1 g of dried thalli from a Whirl-Pak bag was weighed and then washed by dipping in autoclaved, 0.2 μm-filter-sterilized, deionized water, followed by grinding into pieces using sterilized mortars and pestles. Total lichen-derived DNA was extracted from the lichen pieces according to the instructions stated in the bead-beating method [31] using ISOIL for the beads beating kit (Nippon Gene, Tokyo, Japan). Precipitation of DNA was done with 100% ethanol, which was facilitated by the addition of Ethachinmate (Nippon Gene, Tokyo, Japan). DNA precipitates were re-suspended in Tris-EDTA buffer (pH 8.0) and assessed for quality and quantity using the NanoDrop 2000c (Thermo Fisher Scientific, Waltham, Massachusetts, USA). The resulting retrieved DNA from the lichen samples had a concentration ranging from 0.7 µg to 10.5 µg per sample, with purity (ratio of absorbance at 260 nm–280 nm) ranging between 1.5 to 1.9. The lichen-derived DNA samples were stored at −20 °C.

### 2.3. Amplification, Cloning, and Sequencing of rRNA Gene-Related Sequences

Phylotypes in this study were generated based on the sequences from dual methods, i.e., the conventional Sanger-sequencing method and the massive parallel sequencing technique using an Illumina MiSeq platform. The latter, MiSeq-sequencing, which is described in detail in Section 2.4, was used for profiling a small segment (V3-V4 region) of the 16S rRNA gene sequence in order to add information to the Sanger-based method of sequencing which was used to describe the chloroplast and cyanobacterial phylotypes. In this section, we describe the procedures involved in the Sanger-sequencing of near-full-length 18S RNA genes and internal transcribed spacer (ITS) region of the rRNA genes of fungal mycobionts and algal photobionts and near-full-length 16S RNA genes of the algae-derived chloroplasts and cyanobacteria.

#### 2.3.1. PCR Amplification of rRNA Gene-Related Sequences

The thalli-derived DNA samples (obtained in Section 2.2) were used for the PCR amplification of near full-length 16S rRNA gene sequences of the chloroplasts and prokaryotes, as well as the fungal and algal near full-length 18S rRNA genes and ITS regions (between small- and large-subunit ribosomal RNA genes), by using a TaKaRa Thermal Cycler Dice (TaKaRa Bio, Kusatsu, Japan). All PCR amplifications were conducted with an initial denaturation at 94 °C for 5 min; 30 cycles of 94 °C 30 s, 56 °C 40 s, and 72 °C for 1 min; and the final elongation at 72 °C for 5 min [32], with thraustochytrid-derived [33] and the below-mentioned *Escherichia coli* TOP10 competent cell-derived DNAs as the eukaryotic and prokaryotic positive controls, respectively, and the non-DNA-added reactions as the negative control.

The eukaryotic universal primers for 18S rRNA gene sequence, EukF and EukR [34], were used for the amplification of the lichen-forming fungal and algal 18S rRNA genes (Table 2) and were sequenced with M13F, M13R, and EuK516F primers [35] to obtain the near-full-length 18S rRNA gene sequences.

The ITS regions were amplified using the primer pair ITS1F and ITS4A [36,37] (Table 2). The 16S rRNA gene sequence of algae-derived chloroplasts, which acts as a marker for algae, as well as prokaryotes (including cyanobacteria), was amplified using the universal primers 27F and 1492R [34] (Table 2).

#### 2.3.2. Cloning and Sequencing of rRNA Gene-Related Sequences

The PCR amplicons of fungal and algal 18S rRNA genes were distinguished electrophoretically by their pre-determined sizes of approximately 2.0 kbp and 1.8 kbp, respectively. The electrophoretic bands on the agarose gels were excised for DNA extraction and re-amplification by PCR, with an aim to perform size-specific cloning followed by sequencing. The PCR amplicons of 16S rRNA genes belonged to the algae-derived chloroplasts, cyanobacterial and non-cyanobacterial prokaryotes were collectively used for cloning and sequencing, however the non-cyanobacterial prokaryotic genes were eliminated later from phylotypic analyses.

The PCR amplicons were inserted into the plasmids, followed by transformation into *Escherichia coli* TOP10 competent cells for constructing the clone libraries by using the TOPO TA Cloning kit (Thermo Fisher Scientific, Waltham, MA, USA). The cloned amplicons were sequenced by the Sanger sequencing method, using the BigDye Terminator v3.1 Cycle Sequencing kit (Thermo Fisher Scientific) on an ABI 3730XL automatic DNA Sequencer (Thermo Fisher Scientific).

### 2.4. Massive Parallel Sequencing of V3-V4 Region of the 16S rRNA Genes From the Algae-Derived Chloroplasts, Cyanobacteria, and Other Bacteria

The hypervariable V3-V4 region of the prokaryotic 16S rRNA gene, consisting of approximately 460 bp, was amplified by PCR to construct the libraries for massive parallel sequencing, or next-generation sequencing, by using the MiSeq sequencer (Illumina, San Diego, CA, USA). The preparation of the 16S rRNA gene library began with PCR amplification using the V3-V4 region-specific primer pair 341F and 806R [38,39] (Table 2), which were tail-tagged with Illumina overhang adaptors and indexing barcodes. The target region was amplified using the Kapa HiFi HotStart ReadyMix PCR kit (Kapa Biosystems, Wilmington, MA, USA) on a Takara PCR Thermal Cycler Dice Touch (TaKaRa Bio, Kusatsu, Japan) under the following conditions: 95 °C for 3 min with the lid heated at 110 °C; 25 cycles of 95 °C for 30 s, 55 °C for 30 s, and 72 °C for 30 s; and a final elongation at 72 °C for 5 min, held at 4°C thereafter.

Successful PCR amplicons were purified with the magnetic bead-based Agencourt AMPure XP (Beckman Coulter, Indianapolis, IN, USA). Using the purified amplicons as templates, the second PCR cycle was performed in order to index the amplicons with the Nextera XT indexing primers of S501-S508 and N701-N712 (Illumina). The same PCR condition was employed, except that 8 cycles were used, instead of the previous 25 cycles. The second PCR amplicons were purified as stated above, equimolarly pooled, and run on an Agilent Bioanalyser 2100 (Agilent Technologies, Santa Clara, CA, USA) for quality analysis. The pooled samples were denatured, diluted, combined with the dual-indexed control bacteriophage PhiX, and sequenced on a MiSeq sequencer using the MiSeq Reagent Kit V3 (Illumina) to obtain paired-end (2 × 300 bp) reads.

### 2.5. Data Analysis and Phylotype Determination

We retrieved and analyzed the DNA sequences being classified as fungi, algae, algal-derived chloroplasts, or cyanobacteria. In this study, the non-cyanobacterial prokaryotic sequences, as well as the non-chloroplast organelle sequences, were eliminated.

The Sanger-generated sequences of the 18S rRNA gene and ITS region were aligned by ClustalW multiple alignment program [40] using the BioEdit sequence alignment editor [41]. After excluding the low-quality sequences, the remaining sequences were manually assembled. Chimeric sequences were checked by tree topology analysis [42]. The resulting sequences were grouped into phylotypes, or operational taxonomic units (OTUs), at a sequence similarity cutoff value of 97% using the CD-HIT program [43,44]. A phylotype sequence was represented by the most abundant variant sequence. The phylotypes determined were searched against the NCBI nucleotide (nt) database using BLAST [45]. The sequences that hit with the fungal and algal sequences were used for further phylogenetic tree construction.

For the 16S rRNA gene data, the Sanger-generated sequences were processed following the same method as described above. After the BLAST search, the chloroplast and cyanobacterial sequences were used for the chimera-check analysis, as outlined below. The MiSeq-generated pair-end reads were assembled using fastq-join [46]. After trimming the barcode and primer sequences, the ambiguous sequences that were <300 nt and had a low average quality score (<25) were removed. The taxonomic assignment of the quality-filtered sequences was processed in the web-based tool (https://www.ezbiocloud.net/contents/16smtp) provided by EzBioCloud [47]; a minor modification from the general data-processing procedure was done, not to eliminate the chloroplast 16S sequence. This tool employs a software called CLcommunity (also referred to as BIOiPLUG; ChunLab, Seoul, Korea). This software has been previously used for analyzing microbial community structures [48,49]. Briefly, the method involves denoising the sequences to remove the errors generated by the sequencing process using DUDE-Seq [50]. After denoising, the sequences were compared against the EzBioCloud 16S database, constructed from the curated 16S rRNA gene sequences using BLAST.

The cut-off value (97%) for species-level identification was set according to the previous study [47]. The other cut-offs for genus or higher taxonomic ranks were set according to Yarza et al. [51]. Chimeras of the sequences that did not match at the species level (≥97%) were checked using the UCHIME program [52] and the EzBioCloud’s chimera-free reference database (https://help.ezbiocloud.net/user-guide/mtp-pipeline/chimera-detection/), and the identified chimeric sequences were removed. In this process, the chimeras in the abovementioned Sanger-generated sequences were also assessed. After taxonomical assignment of the retrieved sequences to the chloroplasts and cyanobacteria, these sequences were clustered using CD-HIT at 97% sequence identity cutoff value in order to obtain the phylotype. Singleton sequences were removed during the phylotype picking process, as described in a previous study [53]. The representative phylotypes in the final data set were searched against the NCBI nucleotide database using BLAST.

The Sanger-generated sequences were deposited in the DDBJ/ENA/GenBank database under the accession numbers LC487916 to LC487925, LC487926 to LC487929, and LC487713 to LC487717, for the 18S rRNA gene, ITS, and 16S rRNA gene phylotypes, respectively. The MiSeq-generated sequence data is available at DDBJ/ENA/GenBank under the BioProject number PRJDB8443. The BioSample numbers are SAMD00175323 to SAMD00175328. The DRA accession number is DRA008580.

### 2.6. Diversity Indices and Phylogenetic Tree Analyses of the Phylotypes

The Simpson diversity index was calculated based on the number of phylotypes that compose a genus as well as the number of sequences that compose a phylotype using the PAST 3.25 software [54].

Phylogenetic trees with the phylotypes and closely related sequences were constructed using MEGA 7 [55]. Multiple sequence alignments and evolutionary analyses were conducted using CLUSTAL W [40]. The evolutionary history of the sequences was inferred using the Maximum Composite Likelihood method [56] based on Kimura 2-parameter distances [57].

Dendrograms and heatmaps were generated based on cluster analyses of sample-specific occurrences of phylotypes by using the Heatmapper [58] with average linkage clustering and Euclidean distance measurement.

## 3. Results and Discussion

### 3.1. Phylotype Diversity

In this study, overall, a total of 116,937 sequences of rRNA operon components, i.e., near full-length 18S and 16S rRNA genes, eukaryotic ITS region between the large and small rRNA genes, and the V3-V4 region of 16S rRNA genes, were obtained from the six lichen samples, which were collected at the six sites within a 50 km-range in East Antarctica (Figure 1; Table 1). As noted above, this study focuses on the sequences affiliated with fungi, algae, algae-derived chloroplasts, and cyanobacteria; other organisms such as the non-cyanobacterial prokaryotic phylotypes were removed from the present report. Similar sequences were grouped into phylotypes, based on the categories of fungi, algae, algae-derived chloroplasts, and cyanobacteria, based on a 97% similarity cutoff value. Singleton sequences, not single phylotypes, in the MiSeq-sequenced V3-V4 region were excluded from the analysis. As a result, a total of 57 phylotypes were generated from an overall 116,937 sequences at a 97% similarity cutoff value, resulting in a phylotypic outlook of the lichen composites with taxa affiliations at the genus and higher levels (Table 3).

A 97% similarity cutoff, or threshold, has been commonly used to generate the prokaryotic phylotypes from 16S rRNA gene sequences [59] despite its intrinsic problems [60]. In a recent study, it was shown that the advanced MiSeq-sequencing of the V3-V4 region of 16S rRNA gene employing the 97% cutoff value was used to generate the phylotypes, which may result in over-merging, i.e., grouping two or more species into one. The use of higher cutoff values such as 98% and 99% would yield more specific phylotypes. However, an estimate of over-merging at 97%, 98%, and 99% cutoffs are 5%–45%, 5%–41%, and 5%–36%, respectively, which may not necessarily be a significant improvement by raising the cutoffs [61]. Despite possible underestimation in the eukaryotic diversity caused by over-merging [62], the 97% cutoff has also been applied to eukaryotic ITS [63] and the 18S rRNA gene [64]. Therefore, the 97% cutoff is reasonably employed in this phylotypic study.

**Figure 2:**
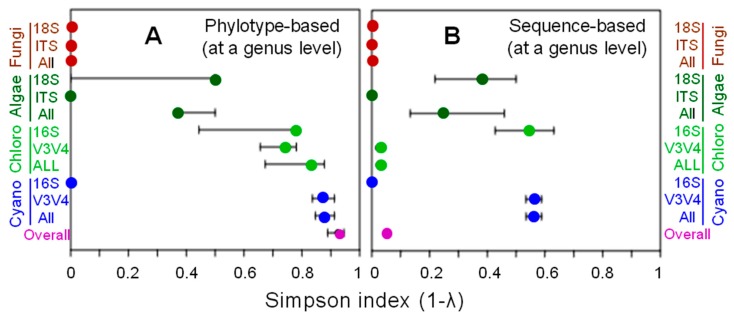
Simpson index (1-λ) as a measure of diversity based on the number of phylotypes that compose a genus (**A**) as well as the number of sequences that compose a phylotype (**B**) of (from top to bottom) lichen-forming fungi, algae, algae-derived chloroplasts, cyanobacteria, and overall (Table 3). Simpson index values with error bars were calculated for: the phylotypes of fungal/algal 18S rRNA gene and ITS region; and of chloroplast/cyanobacterial 16S rRNA gene and V3-V4 region. The actual lower and upper values of the error bars as well as the actual index values are tabulated in Supplementary Table S1.

As the samples were collected from six sites within a relatively narrow range (Figure 1) and used in a one-sample-per-site manner, the phylotypes in the samples were collectively analyzed to calculate the diversity indices such as the Simpson index (1-λ) at a genus level (Figure 2). The calculation was based on the number of phylotypes that compose a genus (Figure 2, A) as well as the number of sequences that compose a phylotype (Figure 2, B). Categories that yielded only one genus, i.e., fungal 18S/ITS, algal ITS, and cyanobacterial 16S, resulted in the Simpson index value of 0. Chloroplasts and cyanobacteria showed relatively high diversity based on the V3-V4 phylotypes. However, based on these sequences, the Simpson index values became lower due to the uneven distribution of the phylotype-composing sequences, as the Simpson index is a measure of species’ richness and evenness. The chloroplast 16S and cyanobacterial V3-V4 resulted in relatively higher diversity than each of the other categories.

### 3.2. Phylotype Composition

From a total of 57 phylotypes, 10 were found to be fungal (consisting of 27 sequences), 4 were algal (14 sequences), 14 were chloroplast-derived (115,406 sequences), and 29 were cyanobacterial (1490 sequences), as summarized in Table 3 and Table 4. Further, 10 fungal phylotypes were affiliated with the representative lichen-forming genus *Umbilicaria*, and 4 algal phylotypes were affiliated with the two representative lichen-hosted green algal genera of *Trebouxia* and *Coccomyxa*. In contrast, the 14 chloroplast phylotypes were affiliated with 10 green algal genera comprising *Coccomyxa*, *Trebouxia*, *Myrmecia*, *Microthamnion*, *Planctonema*, *Ettlia*, *Chloroidium*, *Neglectella*, *Picochlorum*, and *Edaphochlorella*, which may represent diverged lineages, or cryptic diversity, in the chloroplasts [60]. In addition, 29 cyanobacterial phylotypes were affiliated with 13 genera, including the most commonly known cyanobiont genus *Nostoc* [2], along with unclassified ones, which may suggest high variations in the cyanobacterial species that form associations with lichens, as well as opportunistic associations.

Compared to the Sanger-based sequences of near full-length 18S and 16S rRNA genes and the ITS region, the MiSeq-sequences of the V3-V4 region were abundant in numbers as the V3-V4 region, in general, is a part of the 16S rRNA gene and, therefore, some of the retrieved V3-V4 sequences are expected to show a 100% match with those of the 16S rRNA gene sequences, which were amplified separately but were from the same DNA source. However, only few 100% matches were detected, possibly because of the differences in amplification regions (targeted sequences and used primers), presence or absence of the cloning step, and sequencing methodologies. Although the exact reason for the rare occurrence of 100% matches is not clear yet, mismatches between Sanger- and MiSeq-sequencing has become a growing issue in studies involving fungal pathogens [65] and lichen photobionts [9]. According to Paul et al. [9], Sanger sequencing generally failed when the second most abundant photobiont exceeded 30% of the total MiSeq reads in a sample. The second most abundant V3-V4 phylotype in each sample, of either chloroplasts or cyanobacteria, did not exceed 30% of the total reads of the sample (Table 4) and, therefore, the conclusion highlighted by Paul et al. [9] did not apply to this study.

Based on the traditional Sanger-sequenced 18S and 16S rRNA genes and the ITS regions, all lichens except sample 3-2 showed bipartite fungal–algal *Umbilicaria*–*Trebouxia* partnerships (Table 4). Multiple phylotypes per sample were detected on the fungal side, in contrast to a single phylotype (from a single sequence) on the algal side, suggesting that partnerships are established with a specific photobiont. In contrast, sample 3-2 showed tripartite fungal–algal–cyanobacterial partnerships such as *Umbilicaria*–*Trebouxia/Coccomyxa*–*Leptolyngbya* (Table 4), with one genus (composed of six phylotypes) on the fungal side and two genera (composed of three phylotypes from nine sequences) on the algal side, suggesting plasticity in partnerships. These relationships appeared more plastic when viewed based on the V3-V4 sequences. Even the least plastic sample 2-2 with four fungal, one algal, and three cyanobacterial phylotypes (total of seven members) yielded eight chloroplast phylotypes (Table 4). One algal partner for a fungus may not necessarily mean a fixed specific partnership, considering that chloroplast diversity may contribute to the enhanced adaptability of the chloroplast-hosting algae as well as the algae-hosting lichens [66]. However, this result is based only on the six samples of a single lichen genus *Umbilicaria* from limited area coverage and, thus, should not be extended to lichens in other taxa or habitats.

#### 3.2.1. Fungal Phylotypes

**Figure 3:**
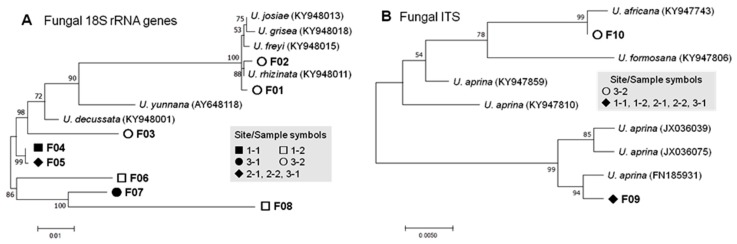
Phylogenetic trees of 18S rRNA genes (**A**) and ITS regions (**B**) of the lichen-forming fungal phylotypes and related *Umbilicaria* species (with accession numbers) generated by the Maximum Composite Likelihood method. The tree with the highest log likelihoods (−3645.00 and −896.60 for A and B, respectively) is shown. The percentage of trees in which the associated taxa clustered together is shown next to the branches. The tree is drawn to scale, with branch lengths measured in the number of substitutions per site. Phylotype codes correspond to those shown in Table 4.

Fungal phylotypes were affiliated with four species of *Umbilicaria*, the representative genus of the rock tripe lichens (Table 3 and Table 4; Figure 3). Three 18S rRNA gene-based phylotypes (referred to as 18S-based phylotypes from hereinafter) were the most closely related to *U. rhizinata* (only in the sample 3-2), which occur predominantly on the high mountains such as the Himalayas and on the King George Island in the maritime Antarctic [67,68]. The other 18S-based phylotype was related to *U. decussate* (in all samples), which is known to occur in East Antarctica [69].

The ITS-based phylotypes were the most closely related to the Iranian strain of *U. aprina* [70] and probably an Afro-Eurasian strain of *U. africana* [71]; both species were recorded as lichens in East Antarctica [72,73]. Usually the ITS regions and rRNA genes of different fungal species are variably amplified by PCR [74], which is likely the case in this study.

#### 3.2.2. Algal Phylotypes

**Figure 4:**
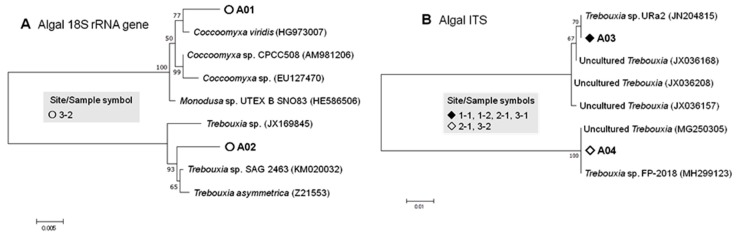
Phylogenetic trees of 18S rRNA genes (**A**) and ITS regions (**B**) of the lichen-associated algal phylotypes and related green algal species (with accession numbers) generated by the Maximum Composite Likelihood method. The tree with the highest log likelihoods (–3335.88 and –1105.35 for A and B, respectively) is shown. The percentage of trees in which the associated taxa clustered together is shown next to the branches. The tree is drawn to scale, with branch lengths measured in the number of substitutions per site. Phylotype codes correspond to those shown in Table 4.

The 18S-based algal phylotypes were affiliated primarily with a *Coccomyxa viridis* strain, which was isolated in a free-living form from wet sandstone in Germany [75], and with the strain *Trebouxia* sp. SAG 246, which was maintained at the Culture Collection of Algae at the University of Göttingen (SAG, Germany) but is currently not available in the SAG Catalogue (https://sagdb.uni-goettingen.de/). Both the phylotypes were detected only in sample 3-2 (Table 3 and Table 4; Figure 4).

The ITS-based phylotypes were affiliated with *Trebouxia* sp. URa2, which was recorded as a photobiont of the lecideoid lichen *Lecidea andersonii* in Queen Maud Land, East Antarctica [26] and with *Trebouxia* sp. FP-2018, hosted by the presumed Mediterranean-endemic lichen *Lasallia hispanica* [9]. The phylotype related to *Trebouxia* sp. URa2 was found only in samples 1-1, 1-2, 2-1, and 2-2, which were collected from areas 1 and 2, i.e., Langhovde Hills and Skarvsnes Foreland, respectively. On the other hand, the phylotype related to *Trebouxia* sp. FP-2018 was found only in samples 3-1 and 3-2 from area 3, Skallen Hills. It is not clear whether the separate distribution was due to the local biogeography or coincidental methodical sample-to-sample variation.

An Antarctic-wide survey showed 141 ITS-based algal phylotypes, all affiliated with the genus *Trebouxia*, from 12 saxicolous lecideoid lichen species [26]. In contrast, three *Trebouxia* and one *Coccomyxa* phylotypes from 10 *Umbilicaria* phylotypes were found in this study. The low algal diversity might be because of the narrow area coverage resulting in less diverse geographic features and to the nature of the *Umbilicaria* mycobionts.

#### 3.2.3. Chloroplast Phylotypes

**Figure 5:**
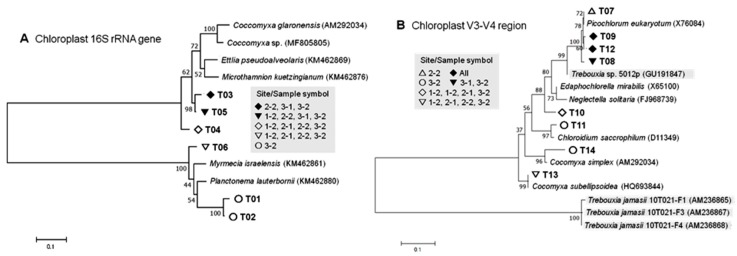
Phylogenetic trees of 16S rRNA genes (**A**) and V3-V4 regions (**B**) of the lichen-associated algae-derived and related chloroplast phylotypes (with accession numbers) generated by the Maximum Composite Likelihood method. The tree with the highest log likelihoods (−6819.36 and −1396.40 for A and B, respectively) is shown. Four known *Trebouxia*-hosted chloroplast sequences are lightly shaded. The percentage of trees in which the associated taxa clustered together is shown next to the branches. The tree is drawn to scale, with branch lengths measured in the number of substitutions per site. Phylotype codes correspond to those shown in Table 4.

Chloroplast phylotypes were affiliated only loosely with those hosted by 10 green algal genera, mostly (nine genera) within the class Trebouxiophyceae, but one (T03; *Ettlia*) affiliated at an 87.6% similarity with the class Chlorophyceae (Table 3 and Table 4; Figure 5). The highest similarity of 97.7% was seen for T11 with the chloroplast of *Chloroidium saccharophilum*, a known lichen-hosted green alga [76]. Chloroplasts of the green alga *Coccomyxa* were related mostly to the phylotypes from the sample 3-2, along with a few incidences in the sample 2-2 (Table 4). Another green algal host genus *Trebouxia* was found in all the samples in this study; however, no *Trebouxia*-affiliated phylotypes of chloroplast were detected, probably because of the relative lack of registered chloroplast 16S rRNA gene sequences of the *Trebouxia* species [77]. Additionally, we can speculate that the lineages of *Trebouxia*-hosted chloroplast might have been so diverged that they would be affiliated differently.

Most of the 16S rRNA gene-based phylotypes (hereinafter 16S-based phylotypes) were found in samples 2-2, 3-1, and 3-2; and none in sample 1-1. In contrast, sample 1-1 yielded the second largest number of V3-V4 sequences, T12, affiliated at a 92.8% similarity with the chloroplast of the green alga *Neglectella solitaria* in the class Trebouxiophyceae [78]. The majority of the overall sequences (115,407 against 117,027) were generated from this single phylotype, T12, which was eminently found in all the samples; samples from site 3-2 yielded the largest number. The occurrence of *N. solitaria*, formerly *Oocystis solitaria*, in Antarctica has not been archived till date [79], and it is not a recognized photobiont of *Umbilicaria* and other lichens. It should be noted that the phylotype T12, despite the affiliation with *N. solitaria*, is most likely hosted by *Trebouxia* or *Coccomyxa*. Genotypic inventory of microalgal chloroplasts [66] is needed to identify the *Trebouxia* or *Coccomyxa* chloroplasts that have phylotypic affiliation with *Neglectella*, along with the incoherent phylogenetic positions of the few known *Trebouxia*-chloroplasts (Figure 5).

Other dominant non-*Trebouxia*/*Coccomyxa*-affiliated chloroplast phylotypes, T09 and T05, were affiliated with those of *Edaphochlorella mirabilis* and *Myrmecia israelensis* at a similarity of 92.1% and 88.3%, respectively (Table 4). Occurrences of both species in Antarctica has not yet been known [79], however *M. israelensis* is known to serve as a photobiont of the rock-dwelling Verrucariaceae lichens [80]. However, considering the low similarity values, further phylogenetic discussion might not be meaningful.

The discrepancy between the Sanger- and MiSeq-sequencing for lichen photobionts has multiple causes [9], such as differences in target sequences, primers, cloning protocol, sequencing methods, assembly protocols, alignment algorithms, and databases, which are categorized as artifacts. The biological factors for non-detection of *Trebouxia*-affiliated chloroplast phylotypes may be caused by the diverged lineages of the chloroplast, although it is highly speculative that “chloroplast switching” (which resembles “photobiont switching,” a fungal strategy to increase adaptability of a lichen to an ever-changing environment [24,25]) is hypothesized for the host algae.

#### 3.2.4. Cyanobacterial Phylotypes

**Figure 6:**
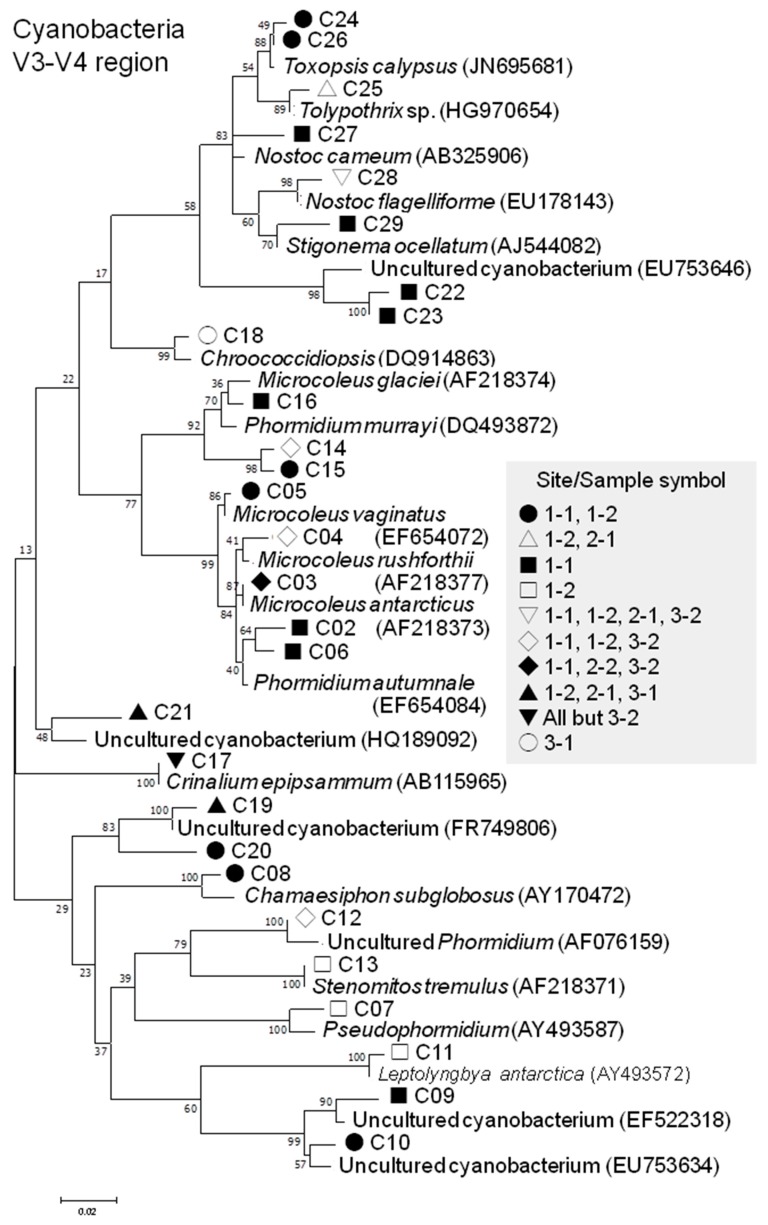
Phylogenetic trees of V3-V4 regions of 16S rRNA gene of the lichen-associated cyanobacterial phylotypes (with accession numbers) generated by the Maximum Composite Likelihood method. The tree with the highest log likelihoods (−3441.61) is shown. The percentage of trees in which the associated taxa clustered together is shown next to the branches. The tree is drawn to scale, with branch lengths measured in the number of substitutions per site. Phylotype codes correspond to those shown in Table 4.

Only one 16S-based cyanobacterial phylotype was detected in sample 3-2 and affiliated with *Leptolyngbya antarctica* (Table 4), which is non-endemic to Antarctic but is widespread in the continent in a free-living form [81]. The occurrence of *L. antarctica*, formerly known as *Phormidium antarcticum*, in the ASPA 141 Yukidori Valley, which corresponds to site 1-1 in this study, was also reported [82]. Another *L. antarctica*-affiliated phylotype, based on the V3-V4 sequence (Table 4; Figure 6), was found in the sample from site 1-2. However, no *L. antarctica*-affiliated V3-V4 phylotype was detected in the sample from site 1-1, which may suggest that *L. antarctica* may be involved in lichen-formation or lichen-association as an opportunistic bystander. The same interpretation would hold true for other cyanobacteria, considering their airborne dispersibility in the Antarctic [83].

*Nostoc* is generally the most commonly known cyanobacterial genus in lichens [84], and two *Nostoc*-affiliated phylotypes (C27 and C28) were detected in *Umbilicaria* lichens in this study. However, the two phylotypes consisted only of 17 sequences, compared with a total of 1490 cyanobacterial sequences (Table 4). The largest number, 953, of the sequences constituted five phylotypes (C03-C05, C14, and C15) affiliated with four *Microcoleus* species: *M. antarcticus*, *M. rushforthii*, and *M. glaciei* of the Antarctic origin [85], along with *M. vaginatus* of which its occurrence in Antarctica has not been recorded yet. It should be noted that *M. vaginatus* occurs in the Arctic Spitsbergen [86] and its phylotypes phylotypes from the substrate of lichens in Navarino Island, the southernmost island of Chile [87], was reported, and its role in the formation of biological soil crust which eventually leads to contact with lichens is well known [88]. Therefore, their predominance in the *Umbilicaria* lichens in this study, particularly in the sample from site 1-1, is notable and may be considered as aiding in providing certain ecological and biogeochemical roles in the lichen and ambient systems. Their predominance as lichen-forming or lichen-associated cyanobacteria would increase the scientific value of the region and the need of monitoring the ASPA 141 Yukidori Valley.

The second most abundant affiliated species was *Toxopsis calypsus* (with the phylotypes C24 and C26), which represents the newly proposed genus and species in 2012, based on a culture from a Greek cave [89]. A phylotype affiliated with *Toxopsis* was found to be abundant in the inland soils of East Antarctica, and its association with moss and lichens was suggested [90].

### 3.3. Phylotypic Profiles of the Studied Samples

The rock tripe lichen samples in this study were collected from three areas over a 50 km range (Langhovde, Skarvsnes, and Skallen, depicted in Figure 1) but were analyzed as one-sample-per-site (*n* = 1) and, thus, do not statistically represent the biogeographic features of the sites.

As stated above, samples 3-1 and 1-1 were distinctly unique in the predominant occurrences of the chloroplast phylotypes affiliated with those of the *Neglectella solitaria* and other green algae and the *Microcoleus*-affiliated cyanobacterial phylotypes, respectively. These phylotypes, as well as the living specimens, have rarely been recorded in East Antarctica, or whole Antarctica, however they were massively detected by the V3-V4-targetted MiSeq, high-throughput sequencing.

**Figure 7:**
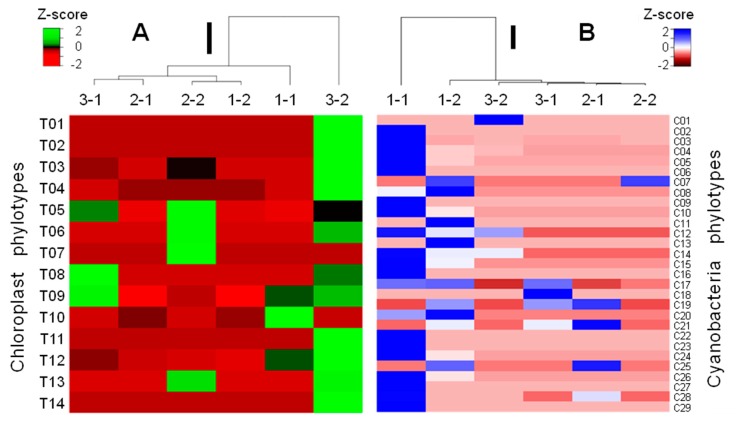
Heatmaps encompassing dendrograms of cluster-analyzed lichen samples based on the phylotype compositions of algae-derived chloroplasts (**A**) and lichen-associated cyanobacteria (**B**). The uniqueness of samples 3-2 and 1-1 is caused by predominant occurrences of specific phylotypes of chloroplast and cyanobacteria, respectively. Phylotype codes correspond to those shown in Table 4. Thick bars beside dendrograms indicate Euclidean distances of the dendrograms as 2000 (A) and 200 (B).

Heatmaps were generated to encompass dendrograms by cluster analysis of the phylotype compositions of chloroplasts and cyanobacteria (Figure 7). The dendrograms revealed the uniqueness of samples 3-2 and 1-1 in the phylotype compositions of chloroplasts and cyanobacteria, respectively. Heatmaps roughly aid in the visualization of the phylotypes contributing to the sample uniqueness (Figure 7). For chloroplast phylotypes, sample 3-2 was characterized by yielding 13/14 chloroplast phylotypes as well as the largest number of V3-V4 sequences of chloroplasts, with the most abundant phylotype T12 (affiliated at a 92.8% similarity with the chloroplast of *Neglectella solitaria*; Table 4). For cyanobacteria, sample 1-1 was characterized by yielding 21/28 cyanobacterial phylotypes as well as the largest number of cyanobacterial V3-V4 sequences, with five abundant phylotypes C03, C04, C05, C14, and C15 (affiliated at >96.9% similarities with the genus *Microcoleus*; Table 4). Because the *Microcoleus* cyanobacteria may have ecological and biogeochemical functions as stated above, sample 1-1 might represent a hotspot of lichen-microbial interactions.

## 4. Conclusions

In the rock tripe *Umbilicaria* lichens from narrow-ranged sites in East Antarctica, unexpected diversities in the green algal chloroplasts and cyanobacteria were observed. Chloroplast phylotypes were predominated not by the lichen-hosted green algal (*Trebouxia* and *Coccomyxa*) chloroplasts, but by those affiliated with less common green algal chloroplasts, suggesting cryptic diversity inside the microalgal single cells. Cyanobacterial phylotypes of a single sample from an Antarctic Specially Protected Area were dominated not by the common lichen partner *Nostoc*, but by the uncommon *Microcoleus*. The occurrence of these less common lichen-forming components may contribute to phenotypic, as well as genotypic, variability within the rock tripe lichen photobionts.

## Figures and Tables

**Table 1:** List of sampling sites of rock tripe lichen specimens along the east coast of Lützow-Holm Bay, Queen Maud Land, East Antarctica, and the numbers of sampled colonies.

Area	Site/Sample	Longitude	Latitude	No. of Sampled Colonies
1. Langhovde Hills	1-1	69˚14′38.9” S	39˚44′59.3” E	1
	1-2	69˚15′38.0” S	39˚47′03.7” E	1
2. Skarvsnes Foreland	2-1	69˚27′37.2” S	39˚47′15.6” E	1
	2-2	69˚29′27.0” S	39˚36′09.6” E	1
3. Skallen Hills	3-1	69˚40′22.0” S	39˚24′11.1” E	2
	3-2	69˚40′28.4” S	39˚24′14.0” E	1

Six sites in three discrete areas were set. One sample per one site was used in this study and, thus, sample designations are the same as the designations of the corresponding sites.

**Table 2:** List of primers used for PCR amplification of target sequences.

Target Sequence	Primer Designation	Forward/Reverse	-mer	5′ → 3′	Expected Product Size	Ref
18S rRNA gene	EukF	F	21	AACCTGGTTGATCCTGCCAGT	Fungi, 2.0 kbp Algae, 1.8 kbp	[34]
	EukR	R	21	TGATCCTTCTGCAGGTTCACC		
Eukaryotic internal transcribed spacer (ITS)	ITS1F	F	22	CTTGGTCATTTAGAGGAAGTAA	800 bp	[36]
	ITS4R	R	20	TCCTCCGCTTATTGATATGC		[37]
16S rRNA gene	27F	F	20	AGAGTTTGATCCTGGCTCAG	1.5 kbp	[34]
	1492R	R	19	GGTTACCTTGTTACGACTT		
V3-V4 of 16S rRNA gene	341F	F	17	CCTACGGGNGGCWGCAG	460 bp	[38]
	806R	R	21	GACTACHVGGGTATCTAATCC		[39]

Primers for sequencing purpose [31] are mentioned in the text. “W” in the 341F sequence indicates the degenerated nucleotides of A or T; “H” in the 806R sequence indicates the degenerated nucleotides of A or C or T; and “V” also in the 806R sequence indicates the degenerated nucleotides of A or C or G.

**Table 3:** Numbers of phylotypes and phylotype-composing sequences affiliated with the most closely related taxa by BLAST.

		Class	Order	Family	Genus	Phylotype Number (%) Codes	Sequence Number (%)
Fungi	18S	Lecanoromycetes	Umbilicariales	Umbilicariaceae	*Umbilicaria*	8 (14.0%) F01–F08	21 (0.018%)
	ITS	Lecanoromycetes	Umbilicariales	Umbilicariaceae	*Umbilicaria*	2 (3.5%) F09, F10	6 (0.005%)
Algae	18S	Trebouxiophyceae	Trebouxiales	Coccomyxaceae	*Coccomyxa*	1 (1.8%) A01	2 (0.002%)
				Trebouxiaceae	*Trebouxia*	1 (1.8%) A02	6 (0.005%)
	ITS	Trebouxiophyceae	Trebouxiales	Trebouxiaceae	*Trebouxia*	2 (3.5%) A03, A04	6 (0.005%)
Chloro-plasts	16S	Trebouxiophyceae	Trebouxiales	Coccomyxaceae	*Coccomyxa*	2 (3.5%) T01, T02	4 (0.003%)
				Trebouxiaceae	*Myrmecia*	1 (1.8%) T05	60 (0.051%)
			Microthamniales	Microthamniaceae	*Microthamnion*	1 (1.8%) T04	13 (0.011%)
			Chlorellales	Oocystaceae	*Planctonema*	1 (1.8%) T06	3 (0.003%)
		Chlorophyceae	Chlamydomonadales	incertae sedis	*Ettlia*	1 (1.8%) T03	13 (0.011%)
	V3-V4	Trebouxiophyceae	Chlorellales	Chlorellaceae	*Chloroidium*	1 (1.8%) T11	3 (0.003%)
				Oocystaceae	*Neglectella*	1 (1.8%) T12	114,009 (97.496%)
				incertae sedis	*Picochlorum*	1 (1.8%) T07	2 (0.002%)
			Prasiolales	Prasiolaceae	*Edaphochlorella*	3 (5.3%) T08–T10	1216 (1.040%)
			Trebouxiales	Coccomyxaceae	*Coccomyxa*	2 (3.5%) T13, T14	83 (0.071%)
Cyano-bacteria	16S	Cyanophyceae	Synechococcales	Leptolyngbyaceae	*Leptolyngbya*	1 (1.8%) C01	1 (0.001%)
	V3-V4	Cyanophyceae	Oscillatoriales	Oscillatoriaceae	*Phormidium*	3 (5.3%) C02, C06, C12	55 (0.047%)
				Microcoleaceae	*Microcoleus*	5 (8.8%) C03–C05 C14, C15	953 (0.815%)
					*Pseudophormidium*	1 (1.8%) C07	2 (0.002%)
				Coleofasciculaceae	*Wilmottia*	1 (1.8%) C16	34 (0.029%)
				Gomontiellaceae	*Crinalium*	1 (1.8%) C17	57 (0.049%)
			Synechococcales	Chamaesiphonaceae	*Chamaesiphon*	1 (1.8%) C08	5 (0.004%)
				Leptolyngbyaceae	*Leptolyngbya*	1 (1.8%) C11	2 (0.002%)
					*Stenomitos*	1 (1.8%) C13	2 (0.002%)
			Chroococcidiopsi-dales	Chroococcidiopsi-daceae	*Chroococcidiopsis*	1 (1.8%) C18	4 (0.003%)
			Nostocales	Nostocacea	*Nostoc*	2 (3.5%) C27, C28	17 (0.015%)
				Godleyaceae	*Toxopsis*	2 (3.5%) C24, C26	204 (0.174%)
				Tolypothrichaceae	*Tolypothrix*	1 (1.8%) C25	12 (0.010%)
				Stigonemataceae	*Stigonema*	1 (1.8%) C29	31 (0.027%)
			Uncultured / unclassified			7 (12.3%) C09, C10, C19–C23	111 (0.094%)

Phylotypes were grouped at a 97% similarity cutoff value for near full-length 18S rRNA genes and eukaryotic ITS regions of lichen-forming fungi and algae, and near full-length and partial V3-V4 region of 16S rRNA genes of algae-derived chloroplasts and cyanobacteria. Relative abundances (% of total 57 phylotypes or 116,937 sequences) are shown in the parentheses.

**Table 4:** Distribution of the numbers of phylotype-composing sequences (overall total 116,937) in lichen samples.

	Seq:	Code	Closest sequence			Area 1 Langhovde		Area 2 Skarvsnes		Area 3 Skallen		*Sum*
			Organism	Accession no.	Similarity (%)	1-1	1-2	2-1	2-2	3-1	3-2	
**Fungal**	18S	F01	*Umbilicaria rhizinata*	KY948011	99.6	0	0	0	0	0	2	*2*
		F02	*U. rhizinata*	KY948011	99.7	0	0	0	0	0	2	*2*
		F03	*U. rhizinata*	KY948001	99.2	0	0	0	0	0	1	*1*
		F04	*U. decussata*	KY948001	99.2	3	0	0	0	0	0	*3*
		F05	*U. decussata*	KY948001	97.1	0	1	4	3	1	0	*9*
		F06	*U. decussata*	KY948001	93.9	0	1	0	0	0	0	*1*
		F07	*U. decussata*	KY948001	95.1	0	0	0	0	1	0	*1*
		F08	*U. decussata*	KY948001	91.0	0	1	0	0	1	0	*2*
	ITS	F09	*U. aprina*	FN185931	99.5	1	1	1	1	1	0	*5*
		F10	*U. africana*	KY947743	98.7	0	0	0	0	0	1	*1*
	***Fungal total***			***10 phylotypes***		*4*	*4*	*5*	*4*	*4*	*6*	***27***
**Algal**	18S	A01	*Coccomyxa viridis* Trebouxiophyceae	HG973007	99.7	0	0	0	0	0	2	*2*
		A02	*Trebouxia* sp. SAG 2463	KM020032	99.7	0	0	0	0	0	6	*6*
	ITS	A03	*Trebouxia* sp. URa2	JN204815	100	1	1	1	1	0	0	*4*
		A04	*Trebouxia* sp. FP-2018	MH299127	100	0	0	0	0	1	1	*2*
	***Algal total***			***4 phylotypes***		*1*	*1*	*1*	*1*	*1*	*9*	***14***
**Chloroplasts**	16S	T01	*Coccomyxa glaronensis* Trebouxiophyceae	AM292034	92.8	0	0	0	0	0	3	3
		T02	*Coccomyxa* sp. Trebouxiophyceae	MF805805	90.1	0	0	0	0	0	1	1
		T03	*Ettlia pseudoalveolaris* Chlamydomonadales	KM462869	87.6	0	0	0	2	1	10	13
		T04	*Microthamnion kuetzingianum*Trebouxiophyceae	KM462876	84.6	0	1	1	1	0	10	13
		T05	*Myrmecia israelensis* Trebouxiophyceae	KM462861	88.3	0	1	0	35	14	10	60
		T06	*Planctonema lauterbornii* Trebouxiophyceae	KM462880	88.0	0	0	0	2	0	1	3
	V3-V4	T07	*Picochlorum eukaryotum* Trebouxiophyceae	X76084	81.9	0	0	0	2	0	0	2
		T08	*Edaphochlorella mirabilis* Trebouxiophyceae	X65100	94.8	0	0	0	0	3	1	4
		T09	*Edaphochlorella mirabilis* Trebouxiophyceae	X65100	92.1	212	89	116	158	348	260	1183
		T10	*Edaphochlorella mirabilis* Trebouxiophyceae	X65100	94.8	23	2	3	0	0	1	29
		T11	*Chloroidium saccharophilum* Trebouxiophyceae	D11349	97.7	0	0	0	0	0	3	3
		T12	*Neglectella solitaria* Trebouxiophyceae	FJ968739	92.8	21,375	5772	10,596	7924	14,065	54,277	114,009
		T13	*Coccomyxa subellipsoidea* Trebouxiophyceae	HQ693844	91.6	0	0	0	2	0	3	5
		T14	*Coccomyxasimplex* Trebouxiophyceae	AM292034	95.7	0	0	0	0	0	78	78
	***Chloroplast total***			***14 phylotypes***		*21,610*	*5865*	*10,716*	*8126*	*14,431*	*54,658*	***115,406***
**Cyanobacterial**	16S	C01	*Leptolyngbya antarctica* Synechococcales	AY493590	98.2	0	0	0	0	0	1	*1*
	V3-V4	C02	*Phormidium autumnale* Oscillatoriophycideae	EF654084	93.2	32	0	0	0	0	0	*32*
		C03	*Microcoleus antarcticus* Oscillatoriophycideae	AF218373	99.5	181	0	0	1	0	1	*183*
		C04	*Microcoleus rushforthii* Oscillatoriophycideae	AF218377	99.0	478	34	0	0	0	19	*531*
		C05	*Microcoleus vaginatus* Oscillatoriophycideae	EF654072	99.7	203	16	0	0	0	0	*219*
		C06	*Phormidium autumnale* Oscillatoriophycideae	EF654084	98.1	9	0	0	0	0	0	*9*
		C07	*Pseudophormidium* Oscillatoriophycideae	AY493587	97.6	0	1	0	1	0	0	*2*
		C08	*Chamaesiphon subglobosus*Synechococcales	AY170472	97.4	1	4	0	0	0	0	*5*
		C09	Uncultured cyanobacterium (from Rocky Mountains)	EF522318	98.6	22	0	0	0	0	0	*22*
		C10	Uncultured cyanobacterium (from Spanish stromatolites)	EU753634	98.6	8	1	0	0	0	0	*9*
		C11	*Leptolyngbya atarctica* Synechococcales	AY493572	99.5	0	2	0	0	0	0	*2*
		C12	*Phormidium* sp. Oscillatoriophycideae	AF076159	98.6	7	3	0	0	0	4	*14*
		C13	*Stenomitos tremulus* Synechococcales	AF218371	94.6	0	2	0	0	0	0	*2*
		C14	*Microcoleus glaciei* Oscillatoriophycideae	AF218374	96.9	6	2	0	0	0	2	*10*
		C15	*Microcoleus glaciei* Oscillatoriophycideae	AF218374	97.2	8	2	0	0	0	0	*10*
		C16	*Wilmottia murrayi* Oscillatoriophycideae	DQ493872	98.8	34	0	0	0	0	0	*34*
		C17	*Crinalium epipsammum* Oscillatoriophycideae	AB115965	98.6	17	18	1	4	17	0	*57*
		C18	*Chroococcidiopsis* Chroococcidiopsidales	DQ914863	96.0	0	0	0	0	4	0	*4*
		C19	Uncultured cyanobacterium (from Antarctic Peninsula)	FR749806	99.3	0	4	6	0	4	0	*14*
		C20	Uncultured cyanobacterium (from Antarctic Peninsula)	FR749806	98.8	1	2	0	0	0	0	*3*
		C21	Uncultured cyanobacterium (from Antarctic Dry Valleys)	HQ189092	95.7	0	2	6	0	2	0	*10*
		C22	Uncultured cyanobacterium (from Spanish stromatolites)	EU753646	96.7	27	0	0	0	0	0	*27*
		C23	Uncultured cyanobacterium (from Spanish stromatolites)	EU753646	97.2	26	0	0	0	0	0	*26*
		C24	*Toxopsis calypsus* Nostocales	JN695681	96.7	91	11	0	0	0	0	*102*
		C25	*Tolypothrix* sp. Nostocales	HG970654	98.6	0	5	7	0	0	0	*12*
		C26	*Toxopsis calypsus* Nostocales	JN695681	98.6	76	26	0	0	0	0	*102*
		C27	*Nostoc carneum* Nostocales	AB325906	98.1	4	0	0	0	0	0	*4*
		C28	*Nostoc flagelliforme* Nostocales	EU178143	98.6	8	1	3	0	0	1	*13*
		C29	*Stigonema ocellatum* Nostocales	AJ544082	97.9	31	0	0	0	0	0	*31*
	***Cyanobacterial total***			***29 phylotypes***		*1270*	*136*	*23*	*6*	*27*	*28*	***1490***
**Overall total**				**57 phylotypes**		22,885	6006	10,745	8137	14,463	54,701	**11,6937**

Phylotypes were affiliated with the most closely related sequences/organisms. Phylotypes were grouped at a 97% similarity cutoff value for near full-length 18S rRNA genes (18S) and eukaryotic ITS regions of lichen-forming fungi and algae, and near full-length 16S rRNA genes (16S) and partial V3-V4 region of 16S rRNA genes of algae-derived chloroplasts and cyanobacteria.

## Supplementary Materials

Supplementary materials can be found at https://www.mdpi.com/2076-2607/7/7/203/s1.
