# Supplementary material for: Phylotypic Characterization of Mycobionts and Photobionts of Rock Tripe Lichen in East Antarctica
Source: Microorganisms. 2019 Jul 18;7(7):203. doi: 10.3390/microorganisms7070203 (PMC6681027; doi:10.3390/microorganisms7070203)
Supplement: Supplementary file 1 [file microorganisms-07-00203-s001.pdf]

Supplementary Table 1. Simpson index (1-λ) as a measure of genus-level diversity based on the numbers of phylotypes that compose a genus and the numbers of sequences that compose a phylotype of: lichen-forming fungi, algae, algae-derived chloroplasts, cyanobacteria and overall (Table 3). The index values are shown along with the lower and upper values of the error bars depicted in Figure 2.

Mycobiont/ Photobiont	Target gene/ sequence	Simpson index (1-λ) based on:					
		Phylotypes			Sequences		
		Lower	Index	Upper	Lower	Index	Upper
Fungi	18S	0	0	0	0	0	0
	ITS	0	0	0	0	0	0
	All	0	0	0	0	0	0
Algae	18S	0.00	0.50	0.50	0.22	0.38	0.50
	ITS	0	0	0	0	0	0
	All	0.38	0.38	0.50	0.13	0.24	0.46
Chloroplast	16S	0.44	0.78	0.78	0.43	0.54	0.63
	V3-V4	0.66	0.75	0.78	0.02	0.02	0.02
	All	0.67	0.84	0.88	0.02	0.02	0.03
Cyanobacteria	16S	0	0	0	0	0	0
	V3-V4	0.84	0.87	0.91	0.53	0.56	0.59
	All	0.85	0.88	0.91	0.53	0.56	0.59
Overall		0.89	0.92	0.95	0.05	0.05	0.05
